# A novel technique for insertion of left ventricular assist device in a patient with severely calcified left ventricle apex

**DOI:** 10.1016/j.xjtc.2023.03.006

**Published:** 2023-03-20

**Authors:** Fazal W. Khan, Vivienne Pham, Pankaj Garg, Md Walid Akram Hussain, Si M. Pham

**Affiliations:** a Department of Surgery, Mayo Clinic, Rochester, Minn; b Department of Cardiothoracic Surgery, Mayo Clinic, Jacksonville, Fla

**Figure:**
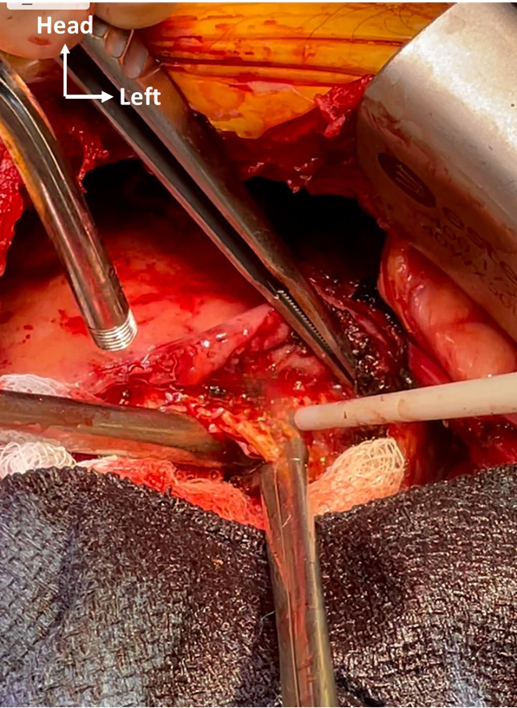
Removal of calcified layer of LV apex with SONOPET device (Stryker Inc) and rongeur.

**Central Message**

We report a novel technique of implanting a HeartMate 3 LVAD (Abbott Laboratories) in a patient with a severely calcified LV apex when thick calcium deposits could not be safely removed.

Changes in left ventricular (LV) morphology, such as apical aneurysm or calcification, present a technical challenge for implantation of a left ventricular assist device (LVAD).^1^, ^2^, ^3^, ^4^ We report a novel technique of implanting a HeartMate 3 LVAD (Abbott Laboratories) in a patient with a severely calcified LV apex.

## Case Presentation

**Figure 1:**
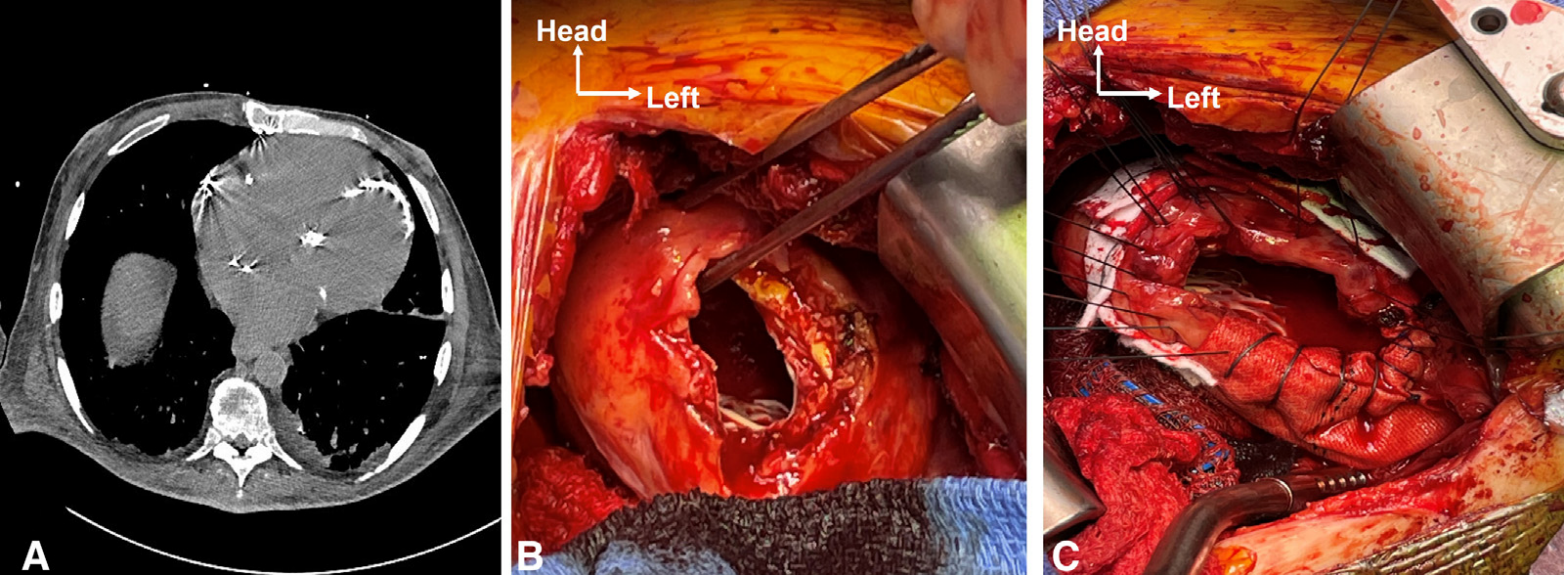
A, Chest computed tomography scan shows calcified LV apex. B, Extensively calcified LV apex with residual rim of calcification attached to a thin layer of myocardium. C, Sutures with pledgets were placed through drilled holes in calcium layer (*upper part*); the thin myocardial rim (*lower part*) was reconstructed with a Dacron patch before placing sutures to secure the HeartMate 3 LVAD (Abbott Laboratories) apical cuff.

**Figure 2:**
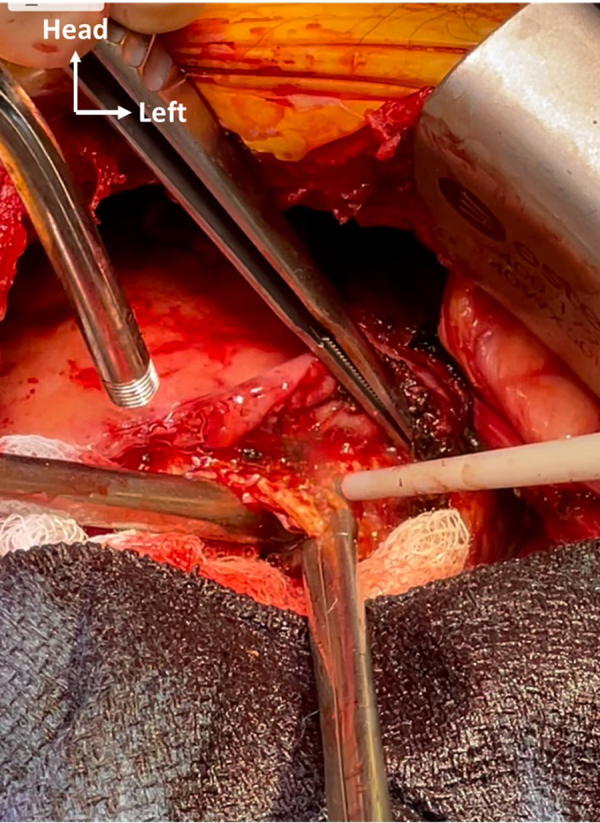
Removal of calcified layer of left ventricular apex with SONOPET device and rongeur.

**Figure 3:**
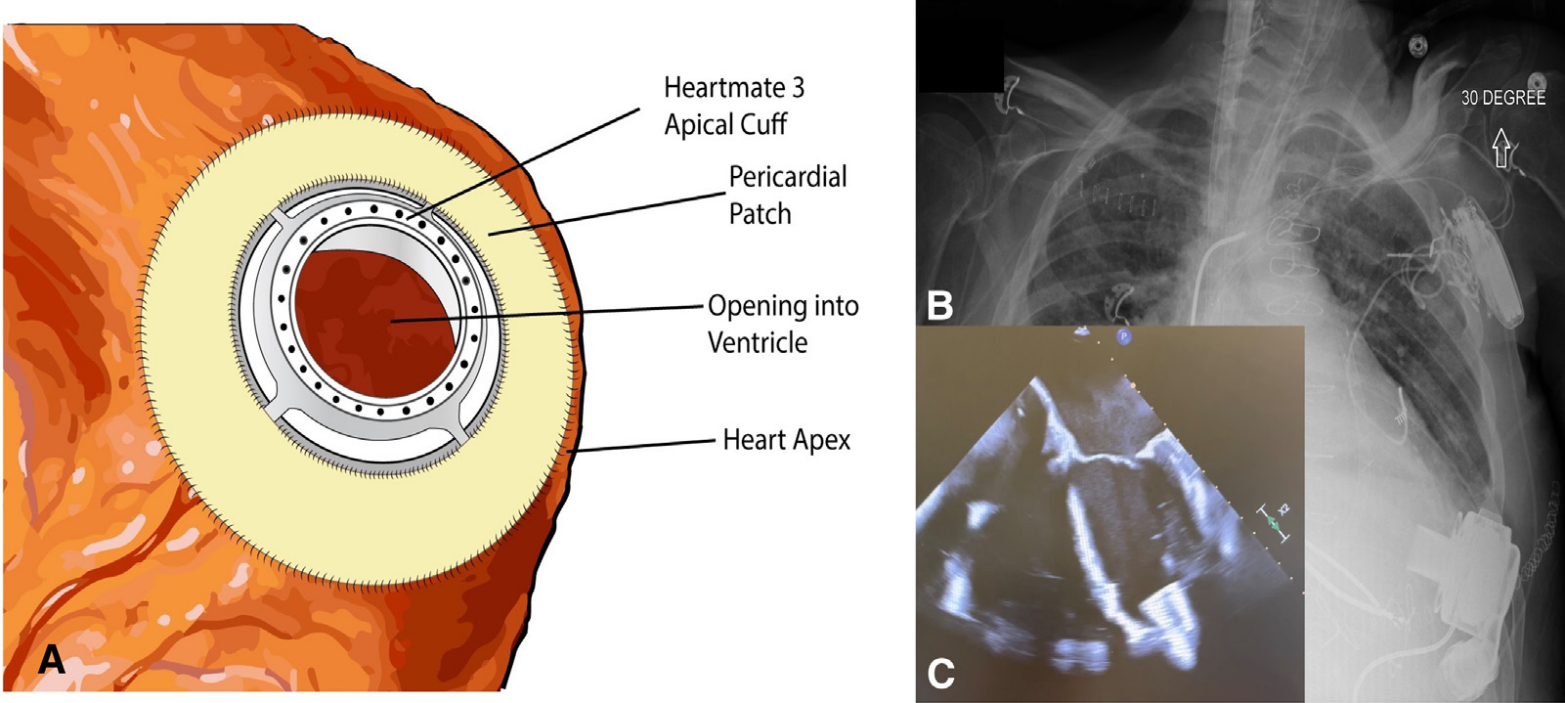
A, Sketch of pericardial patch covering the apical cuff suture lines. Chest radiograph (B) and echocardiogram (C) show good position of the HeartMate 3 inlet in the LV.

The Institutional Review Board provided a waiver for this report. A 64-year-old man with previous coronary artery bypass grafting and epicardial pacing leads presented with cardiogenic shock, requiring a temporary Impella 5.5 LVAD (Abiomed) and a CentriMag pump (Abbott Laboratories) with a Protek Duo cannula (LivaNova) for right ventricular support. A durable LVAD was recommended. The LV had extensive calcification that replaced most of the myocardium and covered the distal third of the septum, the anterior wall, the lateral wall, and the LV apex (Figure 1, *A* and *B* and 2; Video 1). After a redo sternotomy, a venous cannula was inserted, via the right atrium, into the inferior vena cava and connected to the Protek Duo cannula for venous drainage. The aorta was cannulated, cardiopulmonary bypass was initiated, and the Impella device was removed. Because of extensive adhesions, a left thoracotomy at the sixth intercostal space was performed to expose the LV apex. The anteroseptal and lateral aspects of the LV were severely calcified with a thick (1 cm) layer of calcium. Using a combination of heavy scissors, rongeurs, and the SONOPET Ultrasonic Aspirator (Stryker Inc), we removed a rim of calcium to create a circular opening in the LV apex, leaving the thick calcium layer behind (Figure 1, *B* and *C*; Video 1). Because we could not pass suture needles through calcified tissue, we used a dental drill to create holes in the calcified area for passing suture needles (Figure 1, *C*; Video 1). We also reconstructed the thin myocardial layer in the posterior part of the apical opening (Figure 1, *B*) with a Hemashield Dacron patch (Boston Scientific Corp) before placing sutures to secure the apical cuff (Figure 1, *C*). We then sutured a piece of bovine pericardium (Edwards Lifesciences) to the apical cuff and LV apex to cover the suture lines to prevent bleeding and air entrainment. We created a hole in the center of a round piece of bovine pericardium and circumferentially sutured the center rim to the black line on the apical cuff and the outer rim to the epicardium of the LV apex to seal the suture lines (Figure 3, *A*). Successful insertion of the HeartMate 3 inlet and device implantation ensued, with good LVAD inlet position (Figure 3, *B* and *C*). There was good hemostasis of the LV apical site. We continued to support the right ventricle in the perioperative period, using the Protek Duo cannula.

## Discussion

We presented a novel technique of implanting a HeartMate 3 LVAD in a patient with a severely calcified LV apex and LV wall by using commonly available tools to chisel out the calcium and suturing the apical cuff while preserving the LV apical configuration for optimal insertion of the LVAD inlet port. Options for insertion of an intracorporeal LVAD inlet in a severely calcified LV apex include; removing the calcified apex and reconstructing it with Dacron or pericardial patches,^3^ avoiding the calcified area and inserting the inlet port into the diaphragmatic surface^5^ or the anterior lateral wall.^1^ These techniques have limitations, especially when the LV cavity is not large enough, including interference from the mitral valve apparatus, obstruction from the septal wall, and narrowing of LV cavity. The ideal position for the inlet of the HeartMate 3 LVAD is in the LV apex with the inflow port directed toward the mitral valve orifice. Therefore, preservation of the LV apex is best for the insertion of the device.

## Conclusions

By using available surgical tools in most operating rooms, insertion of a durable LVAD in the standard apical configuration is feasible in patients with a severely calcified apex where removal of the calcified myocardium cannot be safely done.

## Supplementary Data

Video 1 Intraoperative video shows the calcified LV apex and the use of the SONOPET Ultrasonic Aspirator (Stryker Inc) to remove calcium. Video available at: https://www.jtcvs.org/article/S2666-2507(23)00074-3/fulltext.
