# Supplementary figures and images for: A novel technique for insertion of left ventricular assist device in a patient with severely calcified left ventricle apex
Source: JTCVS Tech. 2023 Mar 20;19:61–3. doi: 10.1016/j.xjtc.2023.03.006 (PMC10267860; doi:10.1016/j.xjtc.2023.03.006)

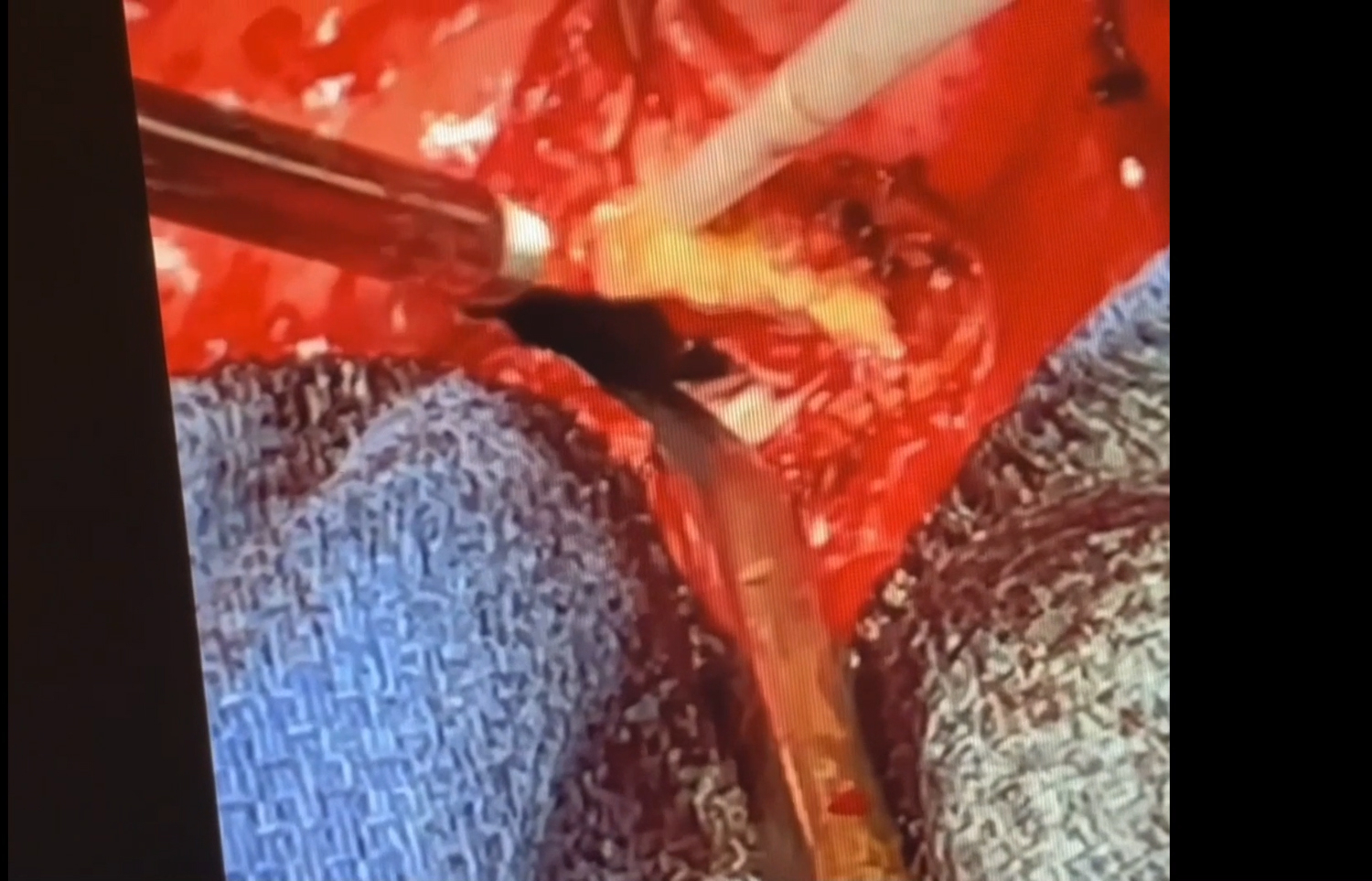

Supplement: Video 1 — Intraoperative video shows the calcified LV apex and the use of the SONOPET Ultrasonic Aspirator (Stryker Inc) to remove calcium. Video available at: https://www.jtcvs.org/article/S2666-2507(23)00074-3/fulltext. [file fx2.jpg]
